# Births, Marriages and Deaths

**Published:** 1848-06

**Authors:** 


From the the Albany Evening Journal.

*Births, Marriages and Deaths.*

Secretary, Office, April 10, 1840.

Dear Sir,—Having just completed the several tables for a report of Births, Marriages, and deaths, to be made to the Legislature, in pursuance of the act of April 28, 1847, in which duty Mr. J. Cuyler was associated with me, I thought it might not be uninteresting to the readers of your paper, to give a few general results gleaned from them. I would, however, remark, that as this is the first report under the law referred to, the reports from the several counties in the State are not as full and complete as could be wished, and unfortunately, owing to an omission in the law, the several cities in the State are, in fact, excluded from its operation, an d noreports have, consequently, been received from them, except a partial one from the city of New York. The law makes it a duty of "The Clerks of the several School Districts of this State, organized according to law, and where there shall be no Clerk, or he shall be incapable of acting, the Trustees or one of them, of such District, shall, annually,on or before the 15th day of January in each year, ascertain, from the most accurate means of information in their power, and report in writing to the Town Clerk of the town or one of the Alderman of the ward."&c. There being no Clerks or Trustees of School Districts in the several cities, there were, consequently, no officers, on whom devolved the duty of collecting the information necessary for this report.

Of the whole number of Marriages, 898 were in the month of January; 841 in February; 820 in March; 612 in April; 701 in May; 730 in June; 942 in July; 770 in August; 1,238 in September; 1,354 in October; 1,151 in November; 1,348 in December.

Of the ages of the persons married, 3,338 were under 20 years; 8,383 between 20 and 25; 4,292 between 25 and 30; 1,300 between 30 and 35; 753 between 35 aud 40; 415 between 40 and 45; 260 between 45 and

50; 198 between 40 and 55; 113 between 55 and 60; 74 between 60 and 65; 110 between 65 and 70; 8 over 70; 3,641 ages not given.

The greatest number of marriages took place during the months of September, October, November, and December. 8,383 were married between the ages of 20 and 25.

Total number of births from January 1, 1847, to January 1, 1848, 35,897; marriages, 11,437; deaths,17,263

Of the whole number of births, 2,327 took place in January; 2,501 in February; 3,079 in March; 3,000 in April; 2,974 in May; 2,860 in June; 3,243 in July; 3,370 in August; 3,301 in September; 3,202 in October; 2,910 in November; 3,016 in December; 83 month not given. Of the sex, 18,722 were males; 16,988 females; 187 not given. Of the complexion, 13,155 white males; 16 580 females; 258 black males; 225 females; 695 complexion not given. Of twins there were 15 pair reported; of illegitimate children there were 118 reported.

The greatest number of births took place in the month of August, viz., 3,370; the smallest number in the month of January, 2,357.

Of the whole number of deaths, 1.032 were in the month of January; 1,128 in February; 1,443 in March; 1,327 in April; 1,239 in May; 1,246 in June; 1,447 in July; 1,870 in August; 1,984 in September; 1,623 in October; 1,335 in November, 1,513 in December; 46 month not given.

Of the sex of those dying 8,613 were males; 8,575 were females; 75 sex not given.

Of the complexion of those dying, 8,157 were white males; 8,121 were white females; 190 were black males; 204 were black females; 591complexion not given.

Of the condition in life of those dying, 3,352 were males married; 2,917 females were married; 5,400 males were unmarried; 4,318 females were unmarried; 1,276 condition in life not given.

The greatest number of deaths took place during the months of August, September, and October.

The ages of those dying were:

| | Males. | Females. | Total. |
|---|---|---|---|
| Under 1 year | 1033 | 833 | 1866 |
| 1 to 2 years | 590 | 584 | 1174 |
| 2 to 5 " | 818 | 723 | 1541 |
| 5 to 10 " | 418 | 407 | 825 |
| 10 to 15 " | 21el | 236 | 447 |
| 15 to 20 " | 276 | 432 | 708 |
| 20 to 25 " | 431 | 537 | 968 |
| 25 to 30 " | 390 | 445 | 835 |
| 30 to 40 " | 535 | 672 | 1207 |
| 40 to 50 " | 494 | 514 | 1008 |
| 50 to 60 " | 502 | 445 | 947 |
| 60 to 70 " | 508 | 503 | 1011 |
| 70 to 80 " | 579 | 546 | 1124 |
| 80 to 90 " | 391 | 294 | 686 |
| 90 to 100 " | 89 | 71 | 160 |
| 100, years and upwards | 15 | 12 | 27 |
| Age not given | .. | .. | 755 |

Of the diseases reported, the following are (amid a multitude of others) the most fatal: Consumption, Dysentery, Inflammation of the Lungs, Dropsy Croup, and Scarlet Fever, of which Consumption is by far the worst.

The average ages of the several occupations of life are as follows: Clergymen, 56 years; Lawyers, 44 do; Physicians, 51 do; Farmers, 57 do. The remainder of the occupations are too numerous, and cannot be stated here, but will be found in full in the reports, when published.

M. L. SCHERMERHORN.

